# Comparative Analysis of Hysterosalpingography and Diagnostic Hysteroscopy Findings in Infertility Evaluation

**DOI:** 10.7759/cureus.81789

**Published:** 2025-04-06

**Authors:** Mahendra G, Sahana M

**Affiliations:** 1 Obstetrics and Gynecology, Adichunchanagiri Institute of Medical Sciences, BG Nagara, IND; 2 Obstetrics and Gynecology, Adichunchanagiri Medical College Bellur Mandya, Mandya, IND

**Keywords:** comparison, diagnosis, hysterosalpingography, hysteroscopy, infertility evaluation

## Abstract

Introduction

Infertility affects most of the women of reproductive age, with uterine and tubal factors contributing significantly. Hysterosalpingography (HSG) and diagnostic hysteroscopy are widely used modalities in infertility evaluation. This study compares the findings of HSG and hysteroscopy to assess their diagnostic accuracy and utility in clinical management.

Materials and methods

A retrospective comparative observational study was conducted at Adichunchungiri Hospital, Karnataka, over six months (April-September 2024). Thirty-nine women aged 23-36 years with primary or secondary infertility underwent both HSG and diagnostic hysteroscopy. HSG assessed tubal patency and gross uterine abnormalities, while hysteroscopy provided direct visualization of intrauterine pathologies. Statistical analysis included sensitivity, specificity, positive predictive value (PPV), and negative predictive value (NPV), with hysteroscopy considered the gold standard for intrauterine pathology.

Results

Bilateral tubal patency was observed in 59% of cases on both HSG and hysteroscopy. HSG demonstrated a sensitivity of 95.83% and specificity of 92.31% for tubal patency. However, hysteroscopy detected additional findings like hydrosalpinx (10.26%), fimbrial cysts (5.13%), and uterine adhesions (7.69%), which were missed by HSG. For uterine abnormalities, HSG had a sensitivity of 70% and specificity of 85.71%, while hysteroscopy provided superior detection of fibroids and septate uterus. Notably, hysteroscopy influenced management decisions, guiding surgical interventions or assisted reproductive techniques in selected cases.

Conclusion

While HSG remains effective for screening tubal patency, diagnostic hysteroscopy offers superior detection of intrauterine pathologies, impacting treatment strategies. Combining both modalities enhances diagnostic accuracy in infertility evaluation.

## Introduction

Infertility is a multifaceted medical condition that affects approximately 10%-15% of women of reproductive age worldwide, posing significant physical, emotional, and psychological burdens on affected couples [1]. It is clinically defined as the failure to achieve pregnancy after 12 months of regular, unprotected sexual intercourse. The etiology of infertility is often multifactorial, involving both male and female factors. Among female causes, tubal and uterine abnormalities, ovulatory dysfunction, and hormonal imbalances are predominant contributors [2]. Tubal factors alone account for nearly 25%-35% of female infertility cases, while uterine pathologies such as fibroids, congenital anomalies, adhesions, and conditions like polycystic ovarian syndrome (PCOS) further contribute to reproductive challenges [3]. Therefore, a thorough evaluation of tubal and uterine integrity is essential for diagnosing the underlying cause and tailoring effective management strategies in infertility treatment.

Several diagnostic modalities are employed to assess the structural and functional status of the female reproductive tract, particularly focusing on the uterus and fallopian tubes. Among these, hysterosalpingography (HSG) and diagnostic hysteroscopy are the most commonly utilized techniques in clinical practice. HSG is a fluoroscopic procedure that involves the introduction of a contrast agent into the uterine cavity to visualize tubal patency and detect gross uterine abnormalities on x-ray imaging [4]. In contrast, diagnostic hysteroscopy is a minimally invasive endoscopic procedure that enables direct visualization of the uterine cavity and tubal ostia, allowing for detailed assessment of intrauterine lesions such as polyps, fibroids, adhesions, and congenital anomalies [5]. Despite the routine use of both modalities, their diagnostic accuracy, sensitivity, and specificity in detecting subtle or complex abnormalities remain subjects of ongoing debate.

HSG, being widely available and relatively cost-effective, has long been a standard screening tool in infertility workups, especially for evaluating tubal patency [6]. One of its key strengths lies in identifying major tubal obstructions and uterine contour abnormalities. However, its limitations include low sensitivity in detecting fine tubal pathologies like hydrosalpinx, fimbrial adhesions, or minor intrauterine lesions such as small polyps or adhesions [7]. Additionally, HSG relies on indirect evidence of tubal patency based on contrast flow, which may lead to false-negative results in cases of partial blockage or peritubal adhesions [8]. Variability in interpretation based on operator skill further affects its diagnostic accuracy [9]. Conversely, hysteroscopy provides direct visualization of the uterine cavity, offering superior sensitivity in detecting subtle intrauterine pathologies and allowing for simultaneous therapeutic interventions when needed [10]. Although it is considered the gold standard for uterine evaluation, hysteroscopy is invasive, requires anesthesia, and may not be as widely accessible in all clinical settings [11].

The diagnostic correlation between HSG and hysteroscopy has been extensively studied, with varying conclusions regarding their concordance. While HSG effectively identifies tubal blockages, hysteroscopy often uncovers additional uterine abnormalities that are missed on HSG, such as intrauterine adhesions or small polyps that can significantly impact fertility [12,13]. Some studies advocate for combining both modalities to improve diagnostic yield and guide targeted management, particularly in cases of unexplained infertility or inconclusive findings on HSG alone [14]. The present study aims to compare the findings of HSG and diagnostic hysteroscopy in women with primary and secondary infertility.

## Materials and methods

The present study was a retrospective comparative observational study conducted to analyze and correlate the findings of HSG and diagnostic hysteroscopy in the evaluation of women with infertility. The study was carried out in the Department of Obstetrics and Gynecology at Adichunchungiri Hospital, BG Nagara, Karnataka, a tertiary care center equipped with advanced radiological and endoscopic facilities. The study was conducted over a period of six months, from April 2024 to September 2024, and included women who underwent both HSG and diagnostic hysteroscopy as part of their infertility workup.

Women aged between 23 and 36 with primary or secondary infertility, who had been attempting conception for at least one year, were considered eligible. Only those who consented to undergo both procedures and had no contraindications to either HSG or hysteroscopy were included. Women with a history of tubal surgery, hysterectomy, known malignancies, severe systemic illnesses, or those who were pregnant or suspected of pregnancy were excluded. A total of 39 women meeting the eligibility criteria were selected through convenience sampling based on referrals for infertility evaluation during the study period.

All participants underwent HSG first, performed by a trained radiologist using a contrast medium to assess tubal patency and detect any gross uterine abnormalities. This was followed by diagnostic hysteroscopy, carried out by an experienced gynaecologist under aseptic precautions, allowing direct visualization of the endometrial cavity and tubal ostia. Findings, such as polyps, fibroids, uterine septum, adhesions, or other intrauterine pathologies, were documented. Both procedures were performed within a short interval to minimize potential changes in uterine or tubal conditions. Relevant demographic and clinical data, including age, duration of infertility, and body mass index (BMI), were also recorded.

**Figure 1:**
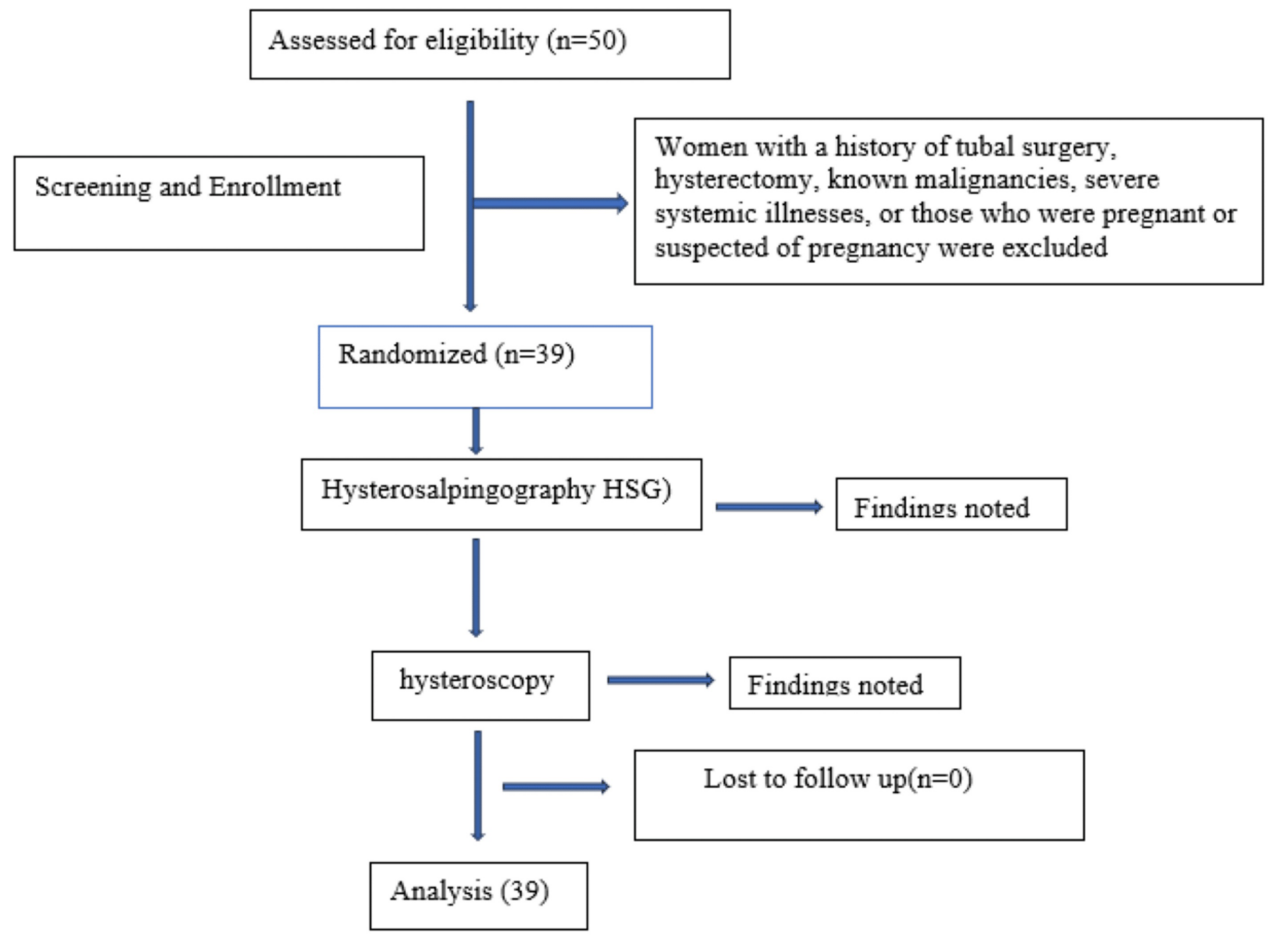
Flow diagram

Data were compiled from procedural findings and medical records, and statistical analysis was performed using descriptive and inferential methods by using SPSS version 26 (IBM Corp., Armonk, NY). The sensitivity, specificity, positive predictive value (PPV), and negative predictive value (NPV) of HSG were calculated using hysteroscopy as the gold standard for intrauterine pathology. Comparative analysis between the two modalities was conducted using the chi-square test, with a p-value of less than 0.05 considered statistically significant. Ethical clearance was obtained prior to the study, and written informed consent was secured from all participants. Patient confidentiality was strictly maintained, and all data were anonymized for analysis in adherence to ethical research standards (Figure 1).

## Results

Demographic characteristics

The study included 39 women with infertility, predominantly in the 31-36-year age group, accounting for 27 participants (69.23%), while 12 participants (30.77%) were between 23 and 30 years old. The majority of the women, 23 (59.00%), had a duration of infertility ranging from one to three years, while 12 (30.77%) reported infertility lasting four to six years, and only four (10.23%) had infertility for more than six years. Primary infertility was observed in 22 women (56.41%) and secondary infertility in 17 women (43.59%). Based on BMI, 23 women (59.00%) had normal BMI (18.5-24.9 kg/m²), nine (23.08%) were overweight, five (12.82%) were obese, and two (5.13%) were underweight (Table 1).

**Table 1:** Demographic characteristics of study participants (n = 39) Frequency and percentages have been written for all variables

Characteristic		Number (%)
Age (years)	23–30	12 (30.77)
	31–36	27 (69.23)
Duration of infertility	1–3 years	23 (59.00)
	4–6 years	12 (30.77)
	>6 years	4 (10.23)
Infertility type	Primary	22 (56.41)
	Secondary	17 (43.59)
BMI (kg/m²)	Underweight (<18.5)	2 (5.13%)
	Normal (18.5–24.9)	23 (59.00)
	Overweight (25–29.9)	9 (23.08)
	Obese (>30)	5 (12.82)

Diagnostic findings of HSG and hysteroscopy

Evaluation of tubal status through HSG showed bilateral tubal patency in 23 women (59.00%), which was consistent with hysteroscopic findings. Unilateral tubal block was reported in seven women (17.95%) on HSG, while hysteroscopy identified six cases (15.38%), with an agreement of 85.71%. Bilateral tubal block was seen in seven women (17.95%) on HSG and six (15.38%) on hysteroscopy, also showing 85.71% agreement. Notably, hydrosalpinx was not detected in any case by HSG but was found in four women (10.26%) by hysteroscopy. Fimbrial cysts were visualized exclusively on hysteroscopy in two women (5.13%). Regarding uterine findings, HSG revealed a normal cavity in 31 women (79.49%), whereas hysteroscopy showed normalcy in only 27 women (69.23%), with an agreement of 87.10%. Fibroids were identified in four women (10.26%) via HSG and five (12.82%) on hysteroscopy. Endometrial polyps were detected equally in three women (7.69%) by both modalities. A septate uterus was identified in one woman (2.56%) by HSG and in two women (5.13%) by hysteroscopy. Additionally, uterine adhesions were detected only by hysteroscopy in three cases (7.69%) (Table 2).

**Table 2:** Diagnostic findings by HSG and hysteroscopy (n = 39) HSG: Hysterosalpingography Frequency and percentage have been written for all variables

Finding	HSG (%)	Hysteroscopy (%)	Agreement (%)
Tubal findings			
Bilateral patent tubes	23 (59.00)	23 (59.00)	100
Unilateral tubal block	7 (17.95)	6 (15.38)	85.71
Bilateral tubal block	7 (17.95)	6 (15.38)	85.71
Hydrosalpinx	0 (0.00)	4 (10.26)	0
Fimbrial cysts	-	2 (5.13)	0
Uterine findings			
Normal uterine cavity	31 (79.49)	27 (69.23)	87.10
Fibroids	4 (10.26)	5 (12.82)	80.00
Endometrial polyp	3 (7.69)	3 (7.69)	100
Septate uterus	1 (2.56)	2 (5.13)	50.00
Uterine adhesions	-	3 (7.69)	0

Diagnostic performance of HSG and hysteroscopy

In assessing the diagnostic performance, HSG demonstrated a sensitivity of 95.83% and specificity of 92.31% for detecting tubal patency, with a PPV of 100% and NPV of 88.89%. For uterine abnormalities, HSG showed a sensitivity of 70.00%, specificity of 85.71%, PPV of 80.00%, and NPV of 77.42%. Hysteroscopy revealed high diagnostic accuracy for fimbrial cysts, with a sensitivity of 85.71%, specificity of 100.00%, PPV of 100.00%, and NPV of 90.00%. Similarly, for detecting fibroids, hysteroscopy had a sensitivity of 80.00%, specificity of 95.24%, PPV of 100.00%, and NPV of 92.31% (Table 3).

**Table 3:** Diagnostic performance of HSG and hysteroscopy PPV: Positive predictive value, NPV: Negative predictive value, HSG: Hysterosalpingography Percentages have been written for all variables

Test parameter	Sensitivity (%)	Specificity (%)	PPV (%)	NPV (%)
HSG				
Tubal patency (bilateral patent)	95.83	92.31	100	88.89
Uterine abnormalities	70.00	85.71	80.00	77.42
Hysteroscopy				
Tubal pathology (fimbrial cysts)	85.71	100.00	100.00	90.00
Uterine abnormalities (fibroids)	80.00	95.24	100.00	92.31

Impact on treatment planning

The findings of both HSG and hysteroscopy had a significant impact on treatment planning. A majority of 25 women (65.00%) with normal tubal and uterine findings were advised to continue with standard infertility treatment. Bilateral tubal block necessitated consideration of tubal surgery or IVF in six women (15.00%). Uterine abnormalities such as fibroids or a septate uterus influenced the management plan toward hysteroscopic surgery or IVF in four cases (10.00%). Hydrosalpinx, detected exclusively on hysteroscopy in three women (7.69%), indicated the need for salpingectomy or IVF. Additionally, fimbrial cysts identified in one case (2.56%) required laparoscopic tubal surgery (Table 4).

**Table 4:** Impact on treatment planning based on HSG and hysteroscopy (n = 39) HSG: Hysterosalpingography

Findings	Effect on treatment	Number (%)
Normal tubal and uterine findings	Continue with standard infertility treatment	25 (65.00)
Bilateral tubal block	Consider tubal surgery or IVF	6 (15.00)
Uterine abnormalities (fibroids, septate)	Consider hysteroscopic surgery or IVF	4 (10.00)
Hydrosalpinx	Consider salpingectomy or IVF	3 (7.69)
Fimbrial cysts	Consider laparoscopic tubal surgery	1 (2.56)

## Discussion

The present study aimed to compare the diagnostic accuracy of HSG and diagnostic hysteroscopy in evaluating uterine and tubal pathologies among women with primary and secondary infertility. Our findings suggest that while HSG is effective as an initial screening tool, especially for assessing tubal patency, diagnostic hysteroscopy provides superior sensitivity and specificity in identifying uterine abnormalities. Tan et al. reported HSG's sensitivity of 85%, specificity of 65%, and overall accuracy of 79.8% for tubal evaluation [15]. Similarly, a study by Panda et al. demonstrated moderate agreement (71.3%) between HSG and hysteroscopy for uterine cavity assessment, reinforcing the complementary nature of these two diagnostic modalities in infertility workup [16].

Despite HSG's utility, disparities in the detection of uterine pathologies were evident. In our study, HSG identified uterine abnormalities in 20.51% of cases, comparable to Bajaj et al., who reported a detection rate of 19% for uterine anomalies, predominantly fibroids and polyps [17]. However, hysteroscopy consistently revealed additional intrauterine abnormalities missed by HSG. In our findings, hysteroscopy detected fibroids (12.82%), polyps (7.69%), septate uterus (5.13%), and intrauterine adhesions (7.69%), highlighting its superior diagnostic capability. Similar observations were made by Panda et al., who reported that hysteroscopy detected additional pathologies in 19.14% of cases HSG failed to identify, including submucosal fibroids, endometrial polyps, and adhesions [16]. These results underscore the limited sensitivity of HSG in uterine pathology detection, further confirmed by Hafizi et al., who reported HSG's sensitivity at merely 38.78% for uterine cavity abnormalities [18].

Regarding tubal patency assessment, HSG demonstrated bilateral tubal patency in 59% of women in our study, consistent with previously reported patency rates ranging from 60% to 65% in infertile populations [15]. Bilateral tubal blockages were observed in 18% of cases, comparable to global statistics that report tubal occlusion rates between 15% and 25% [16]. Interestingly, hysteroscopy confirmed tubal findings in most cases but identified fewer tubal blocks (15.38%) than HSG. This discrepancy could be attributed to the technical limitations of HSG, such as tubal spasms or transient occlusions, potentially leading to false-positive results. Similar findings were reported by Panda et al., who highlighted HSG's tendency to overestimate tubal occlusion due to technical factors [16].

Hysteroscopy's superior diagnostic capabilities, particularly in identifying complex tubal pathologies like hydrosalpinx (10.26%) and fimbrial cysts (5.13%), were significant in this study, conditions that HSG could not detect. These findings are supported by Bajaj et al., who emphasized the superiority of hysteroscopy in identifying subtle tubal and uterine pathologies that often go undetected on HSG [17]. Our study's diagnostic performance metrics reaffirm HSG's strength as a screening tool, with a sensitivity of 95.83% and specificity of 92.31% for tubal patency, which closely aligns with Tan et al.'s report of 85% sensitivity and 65% specificity for HSG [15]. However, the NPV (88.89%) reflects the possibility of missed tubal occlusions, reinforcing the need for confirmatory hysteroscopy, especially in suspected distal tubal pathology or complex intrauterine lesions.

The combination of HSG and hysteroscopy offers a more comprehensive diagnostic approach to infertility evaluation. While HSG remains a cost-effective, non-invasive first-line tool, its diagnostic limitations-particularly for intrauterine abnormalities and subtle tubal lesions-highlight the indispensable role of hysteroscopy. Hysteroscopy demonstrated superior sensitivity (70%) and specificity (85.71%) for uterine pathology in our study, consistent with previous literature positioning it as the gold standard for evaluating the uterine cavity [17]. Moreover, the discrepancies in detecting pathologies such as hydrosalpinx and fimbrial cysts further justify the integration of hysteroscopy into infertility workups to ensure accurate diagnosis and facilitate potential therapeutic interventions in the same setting [18].

This study has several limitations that should be considered when interpreting the findings. The sample size of 39 participants, while adequate for this observational study, may limit the generalizability of the results. More extensive studies with a broader range of participants, including those with different age groups and etiologies of infertility, would provide more robust evidence for the comparison between HSG and hysteroscopy. Additionally, the study did not evaluate the long-term reproductive outcomes following the treatment based on the diagnostic findings, which would offer further insights into the clinical relevance of these diagnostic tools. Future studies with a larger sample size and longitudinal follow-up would help establish a more comprehensive understanding of the diagnostic value of HSG and hysteroscopy in infertility management.

## Conclusions

The present study highlights the complementary roles of HSG and diagnostic hysteroscopy in evaluating female infertility. While HSG is a non-invasive and cost-effective initial screening tool for assessing tubal patency and major uterine abnormalities, it has limitations in detecting subtle intrauterine lesions and certain tubal pathologies. Hysteroscopy provides higher sensitivity and specificity for conditions like fibroids, polyps, septate uterus, adhesions, and hydrosalpinx, and it allows for direct visualization and simultaneous therapeutic interventions. Relying solely on HSG can lead to missed diagnoses and delayed treatment. Therefore, combining HSG with hysteroscopy enhances diagnostic accuracy, improves treatment planning, and can positively impact fertility outcomes in women undergoing infertility evaluation.
